# Charge Transfer Is Promoted by Electronic Heat Exchange in Atoms and Molecules

**DOI:** 10.1021/acs.jpclett.4c03664

**Published:** 2025-02-24

**Authors:** Marco Franco-Pérez, José L. Gázquez

**Affiliations:** † Universidad Nacional Autónoma de México, Cd. Universitaria, Facultad de Química, Ciudad de México 04510, México; ‡ Universidad Autónoma Metropolitana-Iztapalapa, Departamento de Química, Av. San Rafael Atlixco 186, Ciudad de Mexico 09340, Mexico

## Abstract

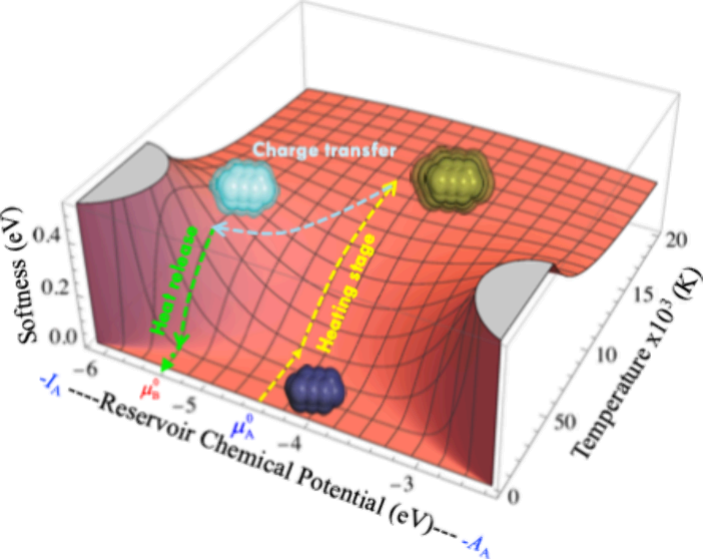

This work compiles almost a decade of theoretical progress in temperature-dependent chemical reactivity theory to introduce the first finite-temperature charge transfer model, predicting fractional electron transfers during chemical interactions. The key insight is that electronic heat drives charge transfer. By analyzing thermodynamic parameters like electronic heat capacity, softness, and chemical potential, the framework explains how species transition from inert to reactive states, where electrons are decorrelated enough to enable charge transfer. A crucial aspect of this model is the role of thermal fluctuations, which governs molecular response functions and facilitates the simultaneous exchange of energy and charge. This model is reduced to a simple linear equation in the chemical potential of the reservoir. When extrapolated, it supports the electrophilicity index, adding a correction term and providing a working formula more influenced by electron affinity. These findings offer new pathways to analyze and predict chemical interactions under the finite temperature regime.

Most chemical interactions are governed by changes in the electronic density of the participating species, that may be interpreted, in many cases, as the transfer of fractional amounts of electronic charge between them. A widely accepted premise for understanding how chemical processes unfold is that “electron-rich species (nucleophiles) donate charge to electron-deficient entities (electrophiles),″ which provides insight into the early stages of many reactions. This statement is also accepted at the local level, nucleophilic moieties of one species donate electronic charge to electrophilic sites of the other. However, the exact mechanisms driving electron transfer, including how electrons are “activated” to engage in fractional charge exchanges, still deserves a deeper explanation. Additionally, accurately quantifying the charge transferred during a chemical process is challenging due to the, up to a certain point, arbitrariness to separate the changes in the electronic density that may be associated with charge transfer, from those associated with polarization. The most used approaches are based on Conceptual Density Functional Theory (C-DFT),^1−13^ which relies on a set of response coefficients or reactivity indicators, to describe the early stages of chemical processes. Despite some well-known mathematical limitations, these models have successfully replicated experimental trends across a range of reactions.^1,14−23^

The notion of “fractional charge” inherently requires an ensemble framework, where fractional electron quantities are computed as averages over a grand ensemble (GE) of species with varying electron counts (e.g., neutral species along with their corresponding anions and cations).^24−27^ Over the past decade, considerable progress has been made in developing a chemical reactivity theory based on the rigorous theoretical framework of the GE at finite temperatures.^26,28−31^ This new approach, known as Temperature-Dependent Chemical Reactivity Theory (τ-CRT), treats molecules as open quantum systems that exchange energy and electrons with their surroundings. By introducing temperature as a new key variable, τ-CRT has provided a more robust framework to study chemical reactivity, address unresolved issues in C-DFT, as well as questions about the electronic structure of chemical systems, including:

1. Differentiability of electronic energy: Even an infinitesimal temperature allows for differentiation of the average electronic energy with respect to the electron’s number to any arbitrary order.^26^
2. Convexity of the Helmholtz free energy potential: The Helmholtz potential may behave as a parabolic function of the electrons number, *N*, supporting the widely used parabolic interpolation of the electronic energy as a function of *N*.^32^
3. Contribution of excited states: Excited states can be naturally incorporated into chemical reactivity calculations.^33^
4. Temperature response coefficients: Variations in temperature yield new response coefficients, including entropy responses, that may offer valuable chemical insights.^34,35^
5. Quantification of charge transfer: The theory enables quantitative evaluation of the electronic charge exchanged between species.^36,37^

Likewise, the τ-CRT framework has allowed the development of precise definitions for a set of position dependent indicators, some of which were inherently ambiguous in C-DFT, emphasizing local hardness,^38−40^ and allowing a detailed rationalization and quantification of a range of chemical processes.^41^ Despite these advances, low-temperature conditions—where most chemical processes occur—introduce several mathematical challenges that may hinder intuitive analysis of chemical reactivity using the τ-CRT framework. The most significant issues include:

1. Second and higher-order chemical reactivity descriptors (e.g., softness) exhibit undesired mathematical behaviors, diverging from widely used zero-temperature (C-DFT) results and complicating the extraction of chemical information.
2. The curvature of the Helmholtz potential vanishes, and it displays and straight-line profile in the electrons number.
3. Contributions from high-energy states are negligible.
4. Descriptors related to temperature variations become insignificantly small.
5. Crucially, predicted fractional charge transfer values approach zero (10^–30^–10^–50^ units and even lesser) for all chemical species, with the only exceptions occurring when the chemical potential of surroundings (of the reservoir) equals the first ionization potential or electron affinity of the species under analysis.^36^
6. It is not possible to naturally extend the chemical potential equalization principle to the low temperature regime.^36^

In this paper, we propose a rigorous and plausible strategy to overcome these mathematical obstacles, offering a renewed framework with new rules and principles for conceiving, interpreting, and quantifying the early stages of chemical interactions.

*Grand ensemble chemical reactivity and charge transfer.* The τ-CRT has been developed under the fundamental framework of the GE at finite temperature for electronic species. Ensemble models are constituted by any chemical species consistent with a unique external potential υ (**r**) (anions, neutral and cations and their excited states). The corresponding GE free energy potential (GP), Ω, takes as input (free) variables the chemical potential of the reservoir, μ_*Bath*_, the temperature *T* and the external potential, *i.e*., Ω ≡ Ω[*T*,μ_*Bath*_,υ (**r**)]. The chemical potential of the reservoir regulates the average amount of electrons in each equilibrium state, while temperature regulates energy transfer.

Temperature dependent chemical reactivity descriptors are defined as response coefficients, similarly than in C-DFT, for instance,^35^
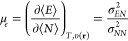
1is the electronic chemical potential at finite temperatures. Quantities in angular brackets are average properties containing the corresponding weighted contributions of all the electronic species constituting the ensemble, *i.e*., ⟨*E*⟩ and ⟨*N*⟩ are the average energy and the average number of electrons, respectively. The quantities σ_*XY*_^2^ = β[⟨*XY*⟩ – ⟨*X*⟩ ⟨*Y*⟩] are thermal fluctuations between the two average properties ⟨*X*⟩ and ⟨*Y*⟩, and β = 1/*kT* where *k* is the Boltzmann constant.

In practice, it is common to appeal to the representative three state ensemble model constituted by the species with *N*_0_, *N*_0_+1 and *N*_0_–1 electrons in their ground state, as their abundance overwhelm their corresponding excited states. Thus, average properties contain the weighted contribution of these three species, while they keep their dependence with the GP free variables. This way, the relative average number of electrons can be expressed as follows,^26^
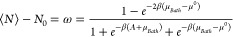
2

μ^0^ = −(*I* + *A*)/2 is the Mulliken chemical potential^42−44^ where *I* and *A* are the vertical first ionization potential and first electron affinity of the species with *N*_0_ electrons, respectively. The quantity ω is known as the fractional charge which by construction is bounded between −1≤ *ω ≤ 1*, and it indicates how ⟨*N*⟩ deviates from the number of electrons of the reference *N*_0_ species.

It is convenient to write ensemble properties in terms of the fractional charge ω, since this way one can analyze how properties are modified during a charge transfer process, and it also allows partial differentiations of average ensemble properties with respect to the electrons number. For instance, the dependence of the (relative) average energy on the fractional charge is given by,^32^
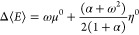
3where Δ⟨*E*⟩ = ⟨*E*⟩–*E*_*N*_0__, η^0^ = *I*–*A* is the denominated Parr and Pearson hardness,^14^ and,
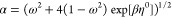
4

Other average properties can be written in a similar fashion, for instance, the relative average density can be written as,^40^
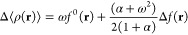
5where  is the Fukui function for the reference state, and (**r**) and (**r**) are the directional Fukui functions for electron accepting and donating processes,^45−47^ while Δ*f* (**r**) = (**r**)–(r) is the working formula for the dual descriptor.^48,49^ Now, taking the partial derivative of eqs 3 and 5 we get,
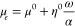
6
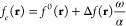
7where *f*_e_(**r**) is the electronic Fukui function. Evidently, μ_e_ = μ^0^ and *f*_e_(**r**) = *f*^0^(**r**) when ω = 0.

It is worth mentioning that like the electronic chemical potential, the general definition of other reactivity descriptors under the finite temperature regime is expressed as the ratio of two thermal fluctuations, (see eq 1). For instance, the general working formula for the electronic Fukui function (partial derivative of the average density with respect to the average electrons number) is *f*_*e*_(**r**) = σ_*Nρ(r)*_^2^/σ_*NN*_^2^,^50^ while the local chemical potential, which spreads μ_*e*_ over the molecular space is defined as μ_*e*_(**r**) = σ_*Eρ(r)*_^2^/σ_*NN*_^2^.^38^ Thermal fluctuations provide insight into how average properties deviate from their equilibrium states due to interactions with a surrounding reservoir. When two average properties, ⟨*X*⟩ and ⟨*Y*⟩, fluctuate together, it indicates a correlation between their variations. For instance, the σ_*EN*_^2^ term represents the coupling between energy changes and variations in electron number, reflecting how electrons in the reservoir carry associated energy (such as kinetic energy) from and toward the system. The ratio of two fluctuation terms, like σ_*EN*_^2^/σ_*NN*_^2^ (representing the electronic chemical potential), measures the system susceptibility to modifications on the external constraints, specifically indicating the energetic sensitivity of a system to fluctuations in its electron population. Such relationships highlight the intrinsic link between chemical reactivity and thermal fluctuations. Extensive interpretations are applicable to other reactivity indicators. For instance, the electronic Fukui function *f*_*e*_(**r**) measures the density sensitivity to variations in the average number of electrons, while the local chemical potential μ_*e*_(**r**) measures the energetic sensitivity to density perturbations due to fluctuations in the electrons number.

It is important to highlight that the electronic chemical potential, μ_*e*_, as described in eqs 1 and (6), and the chemical potential of the reservoir, μ_*Bath*_, are distinct quantities. The electronic chemical potential is an intrinsic property of the system (ensemble), while the reservoir chemical potential is an externally controlled variable, often manipulated by experimentalists. In the context of a highly diluted system, where species 1 (whose electronic states define the ensemble) interacts with a surrounding medium formed by a second species, species 2 represents the environment. Both species are assumed to be in their neutral states (or an *N*-integer electronic state), with species 1 having an electronic chemical potential μ_*e*,1_ = μ_1_^0^, and species 2 (the reservoir) characterized by μ_*Bath*_ = μ_*e*,2_ = μ_2_^0^. The difference between these chemical potentials drives charge transfer between the two species, as outlined in eq 2. Three distinct scenarios for charge transfer can arise: 1) ω = 0 for μ_*e*,1_ = μ_*Bath*_ (no net charge exchange), 2) ω > 0 for μ_*e*,1_ < μ_*Bath*_ (species 1 accepts), and 3) ω < 0 for μ_e,1_ > μ_*Bath*_ (species 1 donates).

Figure 1 illustrates these three charge-transfer scenarios under low and high-temperature conditions for a neutral calcium atom (*I*, *A* = 6.11, 2.37 eV). At low to moderate temperatures (∼10^3^ K), any variation in the reservoir’s chemical potential within the range – *I*_1_>μ_*Bath*_<,–*A*_1_ does not prompt a net exchange of electronic charge. Only when μ_*Bath*_ = −*I*, or μ_*Bath*_ = −*A*, we observe half-unit charge transfers,  and , respectively. These values increase to unity after a slight increment or decrement in μ_*Bath*_, respectively. As the temperature rises beyond ∼10^4^ K, ω nearly becomes a linear function of μ_*Bath*_, allowing the species to exchange significant amounts of electronic charge with surroundings.

**Figure 1:**
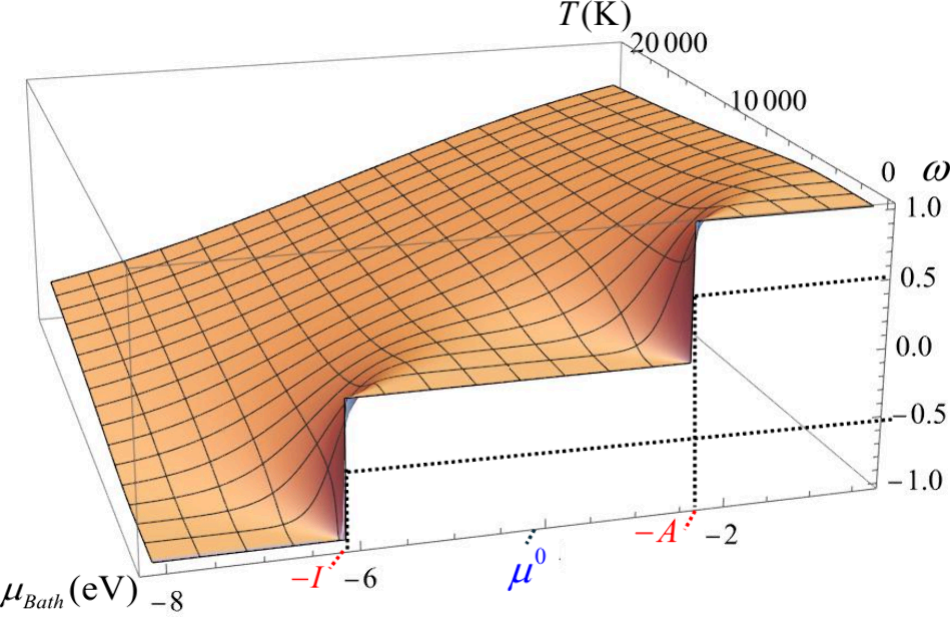
ω vs μ_*Bath*_ profile for the neutral calcium atom (*I*, *A* = 6.11, 2.37 eV) in a range of temperatures.

At equilibrium, one would expect both chemical potentials to equalize, μ_*e*,*eq*_ = μ_*Bath*,*eq*_, indicating that charge transfer has ceased. However, as discussed in the introduction, this equalization principle cannot be fully realized under low-temperature conditions. This is because the electronic chemical potential, μ_*e*_, behaves like a Heaviside function with respect to the reservoir’s chemical potential, μ_*Bath*_, at such temperatures. This step-function behavior prevents full equalization unless μ_*Bath*_ = −*I* or μ_*Bath*_ = −*A*, meaning that at low temperatures, as illustrated in Figure 1, only fractional charge transfers of ±1/2 or the transfer of a full electron can occur.

*Two fundamental electronic temperature thresholds*. Over time, it has become increasingly evident that, within the ensemble framework of electronic systems, the *in situ* temperature experienced by electrons under the influence of an external potential differs significantly from the thermodynamic conditions typically close to room temperature, where most chemical reactions occur. To distinguish these scenarios, the concept of electronic temperature has been introduced, serving as a critical tool for analyzing the unique environmental conditions experienced by electrons within molecules. This concept provides a fundamental basis for modeling, in an averaged manner, how interactions between reacting species perturb electrons and drive them away from their equilibrium states. For instance, a secondary reactant can significantly enhance the kinetic energy of electrons, thereby substantially altering the *in situ* temperature they experience. Electronic temperatures are generally expected to be much higher than thermodynamic temperatures, often exceeding them by one to 2 orders of magnitude. Unless otherwise specified, throughout this manuscript, any reference to temperature in the context of chemical reactivity and charge transfer will exclusively pertain to the concept of electronic temperature.

The anticipated elevated electronic temperature conditions are expected to facilitate chemical reactions through two primary mechanisms. First, excited states become accessible, potentially helping to overcome reaction barriers. Second, overheated electrons would be more prone to engage in charge transfer processes (which may also be favored by the presence of excited states), facilitating the rearrangement of chemical bonding. This last phenomenon is the one considered in the present study. Under this perspective, it is crucial to inspect how to define and compute electronic temperature as well as the consequences of establishing this condition. Two critical electronic temperature thresholds for reactions to occur are here scrutinized.

Let us consider the total variation of the GP with respect to its natural variables,
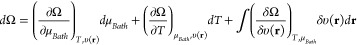
8where,
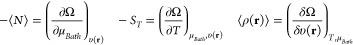
9and *S*_*T*_ is the electronic entropy. Second order coefficients of interest to this study are,
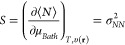
10
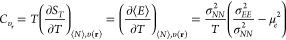
11and,
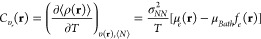
12where *S* is the chemical softness,^51,52^*C*_υ_r__ the electronic heat capacity and *C*_υ_r__(**r**) the local heat capacity.^34,35^ For the representative three state ensemble model eqs 10–12 are simplified as follows,
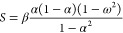
13
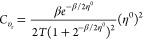
14
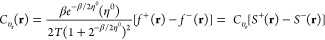
15

Equations 14 and 15 are only valid for ω = 0, at any other condition both heat capacities go to zero in the low temperature limit. It is pertinent to mention that eqs 14 and 15 differ from our previously reported low-temperature formulas^34,35^ due to the presence of a squared term in the denominator of both equations. This term will not be neglected in the current analysis, as we anticipate conditions of elevated electronic temperature where its impact may be relevant.

Defined in terms of thermal fluctuations, these three coefficients (eqs 13–15) are tied to the energy absorbed by the ensemble when external constraints are removed and they provide valuable insights into the mechanisms that originate charge transfer between species. As a thermal fluctuating quantity, softness is a measure of the amount of fractional charge available for electron transfer—the greater the fluctuations, the higher the fractional charge availability. In Figure 2 we show the softness profile of the calcium atom as a function of temperature and electrons number. At low temperatures (like those of chemical interest) *S* → 0 if ω = 0, however it substantially increases, as the average electronic charge becomes fractional. Nonetheless, following the discussion around Figure 1, this substantial increase in softness will only be observed if – *I* ≥.μ_*Bath*_ ≥ – *A*.

**Figure 2:**
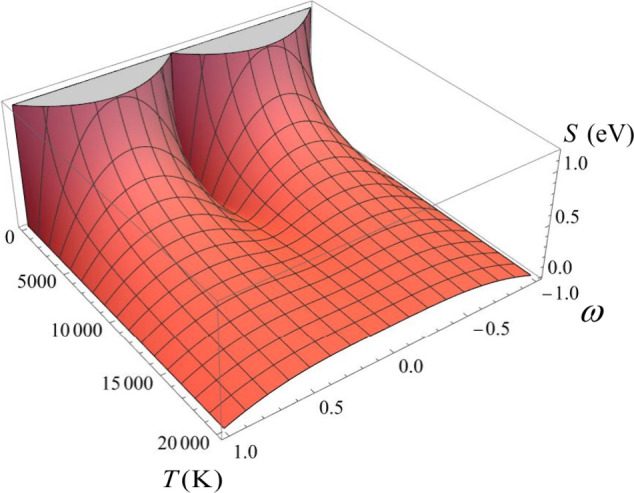
Softness profile as a function of temperature *T* and the fractional charge ω, for the calcium atom.

Heat capacity in turn quantifies the amount of electronic heat that can be exchanged with temperature changes. This heat is confined to the electronic degrees of freedom, as no other degrees are available in our GE model. The local heat capacity arises from treating electrons in molecules as an inhomogeneous gas, where certain regions are more prone to energy exchange than others. As it is shown in the second equality of eq 15, C_υ_r__ is defined as the global heat capacity multiplied by the local function [*S*^*+*^(**r**)–*S*^–^(**r**)], where *S*^*+*^(**r**) = (**r**)/η^0^ and *S*^*–*^(**r**) = (**r**)/η^0^ are the zero-temperature limit definitions of the local softness descriptor for the acceptation and donation process, respectively. Since through its sign the dual descriptor allows one to distinguish between the regions that are more susceptible to an electrophilic attack, Δ*f* (**r**) ≥ 0, from those that are more susceptible to a nucleophilic attack, Δ*f* (**r**) ≤ 0, one can see through the first equality in eq 15 that the sign on the local heat capacity just differentiates if the changes in the electronic density due to changes in the temperature are occurring in the more electrophilic (+) or nucleophilic (−) regions of the molecule.

Setting an adequate electronic temperature condition implies finding a temperature situation where charge transfer is favored the most, properly overcoming the low temperature drawbacks here discussed. An immediate proposal is by finding the *T* that maximizes the chemical softness, increasing the available exchangeable fractional charge. Extremizing eq 13 under the ω = 0 situation we have,^53^
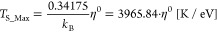
16

Alternatively, one can also search for the extremum of the heat capacity, where the system can gather large amounts of energy and the overall fluctuations of the system are large. In this case, one finds that the maximum of eq 14 is located at,
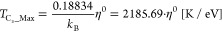
17

Interestingly, both proposals are proportional to the Parr and Pearson hardness, highlighting the close relationship between this parameter and the charge transfer phenomena. While the results from both equations are similar in form and magnitude, the differences remain noteworthy.

Now, let us inspect some ensemble properties at these extremum values. For the chemical softness and the heat capacity we have,
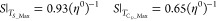
18
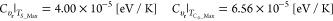
19

Results in eq 18 suggest that the reciprocity relation between *S* and η^0^ proposed in C-DFT, practically holds at T = T_s_Max_. Conversely, chemical species are almost 40 percent harder at *T* = *T*_C_υ__Max_ than in the maximum softness situation. The results in eq 19 are remarkable because they are independent of the electronic structure of the chemical entities, making them universal physical constants across all reacting species. These results impose specific threshold values, the first corresponds to the heat capacity value that an electronic system must reach to attain its maximum softness, while the second establishes a strict boundary condition for the heat capacity applicable to all reacting electronic species.

An additional remarkable insight can be derived from the response coefficient,^30^
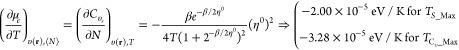
20

Equation 20 reveals that as temperature increases, the acidity of the species is enhanced through a decrease in the electronic chemical potential. Furthermore, the variation in the electronic chemical potential between *T*_C_υ__Max_ and *T*_*S*_Max_ remains nearly constant and appears independent of the specific chemical species.

It is also possible to quantify the amount of heat absorbed by the electronic species going from *T* = 0 to any of the two extremum temperatures under consideration,
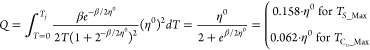
21

As it could be expected, the resulting expression after integration in eq 21 is equivalent to the second term at the right-hand side of eq 3, that is,
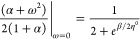
22

This result suggests that the second term at the right-hand side of the energy expression in eq 3 could be seen as the one responsible for heat absorption.

*Overheating electrons resembling a transition phase*. The ability of a molecule to exchange fractional amounts of electronic charge with its surroundings strongly depends on its softness. As shown in Figure 1, before any chemical interaction occurs, when electronic species are in their neutral (integer) states, molecules and atoms are essentially inert to charge exchange, since chemical softness approaches zero at all temperatures of chemical relevance (Figures 2 and 3). Two scenarios could lead to an increase in molecular softness. First, a significant change in the chemical potential of the reservoir could induce charge transfer. However, as discussed, ω remains negligibly small at any condition close to room temperature, only when the reservoir’s chemical potential equals *–I* or −*A* a substantial variation in electron number and in the chemical softness will be observed. From a chemical perspective, this result is inconsistent with the fractional charge transfer phenomena but, more importantly, under the highly diluted consideration, it excludes interactions with species with – *I < μ*_*Bath*_ < – *A*, where μ_*Bath*_ ≡ μ_*e*,2_.

**Figure 3:**
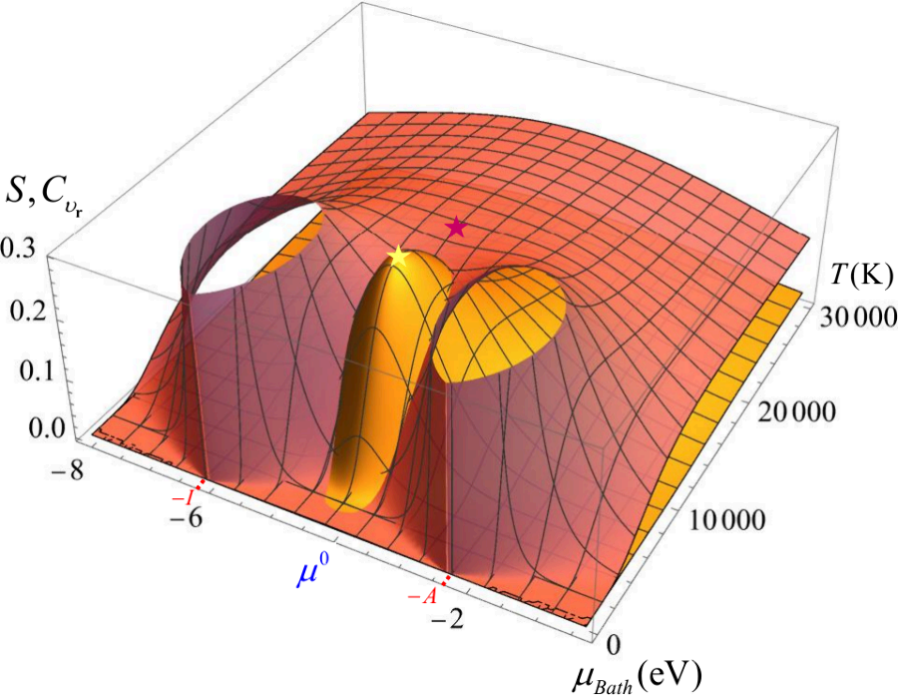
Softness (brick red) and heat capacity (orange) profiles as a function of temperature and the chemical potential of the reservoir, for the calcium atom. For visualization purposes, C_υ_r__ was amplified by a factor of 4500. *S* units are eV, C_υ_r__ units are eV/K. Maximum values of each profile are marked with stars.

As a second alternative, neutral molecules may exchange heat with the environment—other molecules or the reaction medium—heating their electrons and making them more likely to engage in charge transfer. In their neutral state, the heat capacity is small but significantly higher than for the ω ≠ 0 situations, and even more important, as can be inferred from the result in eq 17, the heat capacity is an increasing function of temperature (before *T*_C_υ__Max_), facilitating the successive heat exchange with surroundings. This transferred energy is then partly stored as thermal fluctuations, including increased softness, until the electrons become “ready” for transfer.

Which of the proposed temperature conditions should be considered for charge transfer? To address this, we must first analyze the implications of these values. Figure 3 illustrates the dependence of softness and heat capacity, on temperature and the chemical potential of the reservoir for a neutral calcium atom (for visualization purposes, C_υ_r__ was amplified by a factor of 4500). The detailed working formulas for these calculations are provided in the Supporting Information (Equations ES1 and ES2, respectively). Importantly, all conclusions related to heat capacity when the fractional charge is nonzero were derived using Equation ES2, rather than Equation 14.

The 3D softness profile reveals three peaks, two at the zero-temperature limit when μ_*Bath*_*= −I* and μ_*Bath*_*= −A*, respectively, and a third peak at μ_*Bath*_ = μ^0^, where the fractional charge equals zero, but the temperature is substantially higher. In contrast, the heat capacity exhibits only the critical point predicted by eq 17, also at zero fractional charge. Therefore, at ω = 0, the system undergoes large thermal fluctuations in the region between the two critical temperatures discussed here, as it is illustrated in Figure 3 (according to eq 18, the softness features at *T*_*C*_υ__Max_ are less but comparable to those at *T*_*S*_Max_). Note that after these critical temperatures, both softness and heat capacity approach zero as temperature increases toward infinity, indicating that the temperature range between these critical points is the most relevant for activating electrons via heat exchange.

In Figure 4 we show the C_υ_r__ and ⟨*E*⟩ vs *T* profiles for the calcium atom. It can be observed that when *T = T*_*C*_υ__Max_ there is a small but perceptible change in the slope of the energy profile (green dot). Figures 3 and 4 together depict a typical scenario for second-order phase transition, in this case electrons are being shifted from a chemically inert state to a *reactive phase*. It is important to note that in finite systems, certain phase transitions do not occur precisely at the critical point but slightly beyond it, and all our findings suggest that a transition phase may be occurring within the range *T*_C_υ__Max_ ≤ *T ≤ T*_*S_*Max_. Notably, within the disclosed transition zone, key electronic properties become nearly universal, independently of the microstates of the chemical species under consideration (eqs 19 and (20)), or exhibit a linear dependence on the η^0^ quantity depicting universal coefficients (eqs 16, 18, and 21).

**Figure 4:**
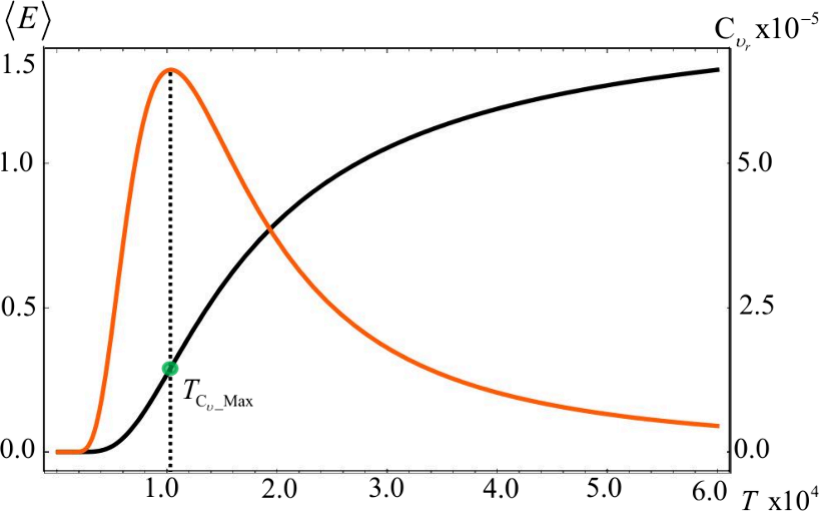
Electronic energy (black) and heat capacity (orange) versus temperature profiles (units in eV). Discontinuous black lines are used to indicate the *T* = *T*_C_υ__Max_ condition. The inflection point in the energy profile is indicated with a green dot.

It is noteworthy that after the *T* = *T*_C_υ__Max_ condition, the system continues absorbing considerable amounts of heat until the maximum softness temperature (*T* = *T*_*s*_Max_) is achieved, allowing other fluctuating properties to also increase (see eq 21), turning the conditions idoneous for charge and energy transfer between chemical species in their neutral state. Consequently, the electronic chemical potential of a second reacting entity (μ_*Bath*_ = μ_2_) can more readily drive the charge transfer process, even if the difference in their electronic chemical potential is relatively small. This analysis suggests that the optimal condition for charge transfer is *T*_*S*_Max_.

It can also be observed that the second term on the right-hand side of the average energy (eq 3), representing the absorbed heat, as previously indicated, is destabilizing. According to eq 22, this term corresponds to the energy required to overcome a barrier, which constitutes a significant fraction of the *I*–*A* gap, approximately 16%. It is known that this gap reflects the degree of electron correlation and orbital localization.^54,55^ Once this energy barrier is surpassed, electrons become partially uncorrelated, allowing them to move more freely as charge carriers. Thus, the destabilizing term in eq 17 represents the energy associated with partial electron decorrelation, *i.e*., *E*_*decorr,ω*=0_ = η^0^ α/2(1+α). These results indicates that *atoms and molecules require energy of about 16% of their gap I*–*A to be sufficiently decorrelated and undergo charge transfer*. This partial loss of correlation, can lead to spatial symmetry breaking, transitioning from an ordered charge-transfer phase to a disordered, inert-electron phase, *i.e*., spatial symmetry facilitates charge transfer. Decorrelation energy has an upper boundary condition equal to 0.664·η^0^ as temperature approaches infinity, indicating that it is not possible to fully decorrelate electrons solely by exchanging heat (a result that also can be inferred from the extremal properties of the heat capacity). As illustrated in Figure 4, the decorrelation term can be seen as, or directly related to, the order parameter of the phase transition. It decreases as the system moves from the ordered to the disordered phase, with a pronounced decline around the phase transition region.

*Extremal principles for cooling down electrons.* Once the phase transition occurs the chemical system is now able to exchange fractional amounts of electronic charge, and the direction of this transference is dictated by the difference in chemical potentials between the system and surroundings. If the maximum hardness principle^56−63^ holds under these circumstances, the softness must decrease as the charge transfer process evolves. This statement should be corroborated by inspecting the concavity of *S* as a function of ω (the corresponding analytical formula is reported in the Supporting Information), *i*.*e*.,
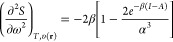
23

Note that at constant temperature, *S* is a concave function of ω if α^3^ > *2e*^*–β*(*I-A*)^. Inspecting eqs 4, 16, 17, and 23 one can conclude that at the *T* = *T*_C_υ__Max_ and *T* = *T*_*S*_Max_ conditions, the softness concavity exclusively depends on the amount of fractional charge exchanged and not on the ensemble microstates. Particularly, for *T* = *T*_*S*_Max_*S* is concave only when |ω| > 0.12, while for *T* = *T*_C_υ__Max_ concavity will be observed for |ω| > 0.16. These results are also valid across any reacting electronic species.

The precedent convexity/concavity analysis indicates that as temperature decreases, the fractional charge range where *S* is convex is broader. In other words, the continuous exchange of fractional charge would be favored if it is accompanied by successive infinitesimal decrease on temperature, leading the ensemble to its highest possible softness state for each increased value of the fractional charge ω. Recall that under the highly diluted consideration, the system is in direct contact with the other reactant as well as the reaction media, and the exchange of heat between them is not hindered. The exchange of electronic charge with the subsequent decrease on temperature will be energetically favored if,
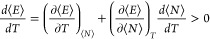
24

Using eqs 1 and 11, conjointly with eq 40 of ref (40), eq 24 simplifies to,
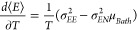
25

By definition, thermal fluctuations in only one average property (σ_*EE*_^2^ for instance) are positive definite while both σ_*EN*_^2^ and μ_*Bath*_ are negative and the product between them is thus positive. For the representative three state ensemble model it is possible to show that σ_*EE*_^2^>(σ_*EN*_^2^)^2^ for – *I*_1_ > μ_*Bath*_ < – *A*_1_ and σ_*EE*_^2^ ≈ (σ_*EN*_^2^)^2^ for μ_*Bath*_ = −*I*_1_,–*A*_1_ at any temperature condition. Thus, eq 25 will be a positive quantity within the *I*_1_ > μ_*Bath*_ < – *A*_1_ range and zero for the μ_*Bath*_ = −*I*_1_,–*A*_1_ situations. For the former case, eq 25 indicates that energy will decrease barely logarithmically with temperature.

The analysis of eqs 23 and 25 suggest that after an infinitesimal exchange of fractional electronic charge, the temperature decreases driving the system toward a state of maximum softness if no other restriction is imposed. This behavior follows what can be termed the *Maximum Softness Path Principle* (MSPP) for charge transfer: “as electronic fractional charge is transferred, energy is released maximizing the softness value of the system, consistently with system restraints”. Importantly, this principle does not conflict with the well-established Maximum Hardness Principle (MHP). At the conclusion of a chemical process, the formed electronic species will assume a new integer *N* (neutral) state, where softness reaches its minimum value (see Figure 2). Although small, the softness of the newly “cooled” species still depends on the η^0^ quantity, and as previously demonstrated, this dependence ensures that the Maximum Hardness Principle holds at the end of the chemical interaction.^62−64^

The Maximum Softness Path Principle (MSPP) stems from the requirement of warming up electrons to initiate charge exchange and defines the optimal conditions for electron flow between a system and its surroundings. However, the MSPP must work in conjunction with another key concept: the Chemical Potential Equalization Principle. While the temperature that maximizes softness sets an ideal environment for electron exchange, it may result in an electronic chemical potential inconsistent with the chemical potential of the reservoir. Consequently, temperature cannot simply decrease without constraint. The system evolves along the maximum softness path as closely as possible, progressing toward a state where the chemical potential equalizes between the system and the reservoir, signaling that charge transfer has ceased. Any excess heat generated during the whole process is then released to other species or the solvent, ensuring energy balance. These coupled principles are known as the finite temperature chemical potential equalization principle or τ-CPEP.

*Gradual electronic charge transfer.* The τ-CPEP offers a practical method for determining the fractional electronic charge exchanged between a species and its environment. This approach requires first identifying the optimal temperature, *T*_*opt*_, at which μ_*e*,*eq*_ = μ_*Bath*,*eq*_. Afterward, the exchanged fractional charge is computed using eq 2, with *T*_*opt*_ and μ_*Bath*_ as inputs. A Python script to perform these calculations is provided in the Supporting Information.

Figure 5a illustrates the relationship between *T*_*opt*_ and μ_*Bath*_ for a neutral bromine atom. The experimental ionization energy (*I*) and electron affinity (*A*) values for bromine are 11.81 and 3.36 eV, respectively. Meanwhile, Figure 5b presents the corresponding softness *S*_*opt*_ at *T*_*opt*_ as a function of μ_*Bath*_, following the τ-CPEP strategy (each *S*_*opt*_ owns an specific *T*_*opt*_ value). For comparison, both figures also display equivalent profiles assuming the MSPP operates freely, represented by the blue curves. Notably, the τ-CPEP and MSPP yield nearly identical results when μ_*Bath*_ is close to μ^0^ (μ^0^ = −7.59 eV for Br). However, as μ_*Bath*_ deviates from μ^0^, the temperature required to meet the μ_*e*,*eq*_ = μ_*Bath*,*eq*_ condition increases significantly compared to that required for the MSPP. Temperature computed from both conditions converge to zero as μ_*Bath*_ approaches *-I* or *-A*. Interestingly, Figure 5b demonstrates that despite the temperature differences, the MSPP holds reasonably well for μ_*Bath*_ values approximately in the range μ_0_ ± 2 eV.

**Figure 5:**
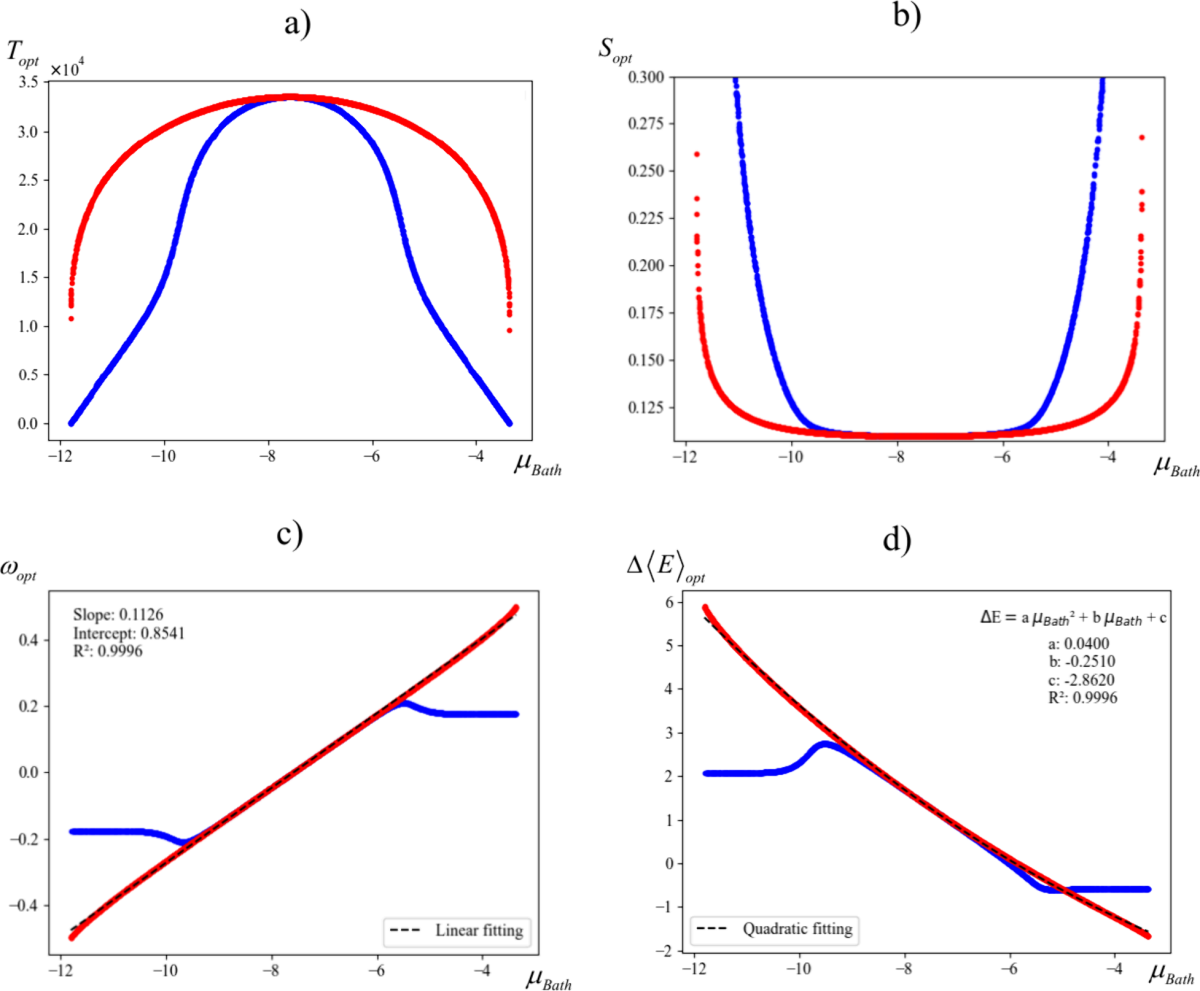
Dependence with μ_*Bath*_ of a) optimal temperature *T*_*opt*_, b) softness *S*, c) charge transferred ω_*opt*_ and d) energy transferred Δ⟨*E*⟩_*opt*_, following the τ-CPEP (red) and the MSPP (blue) principles, for the bromine neutral atom (*I*, *A* = 11.81, 3.36 eV).

Figures 5c and 5d further explore the relationship between μ_*Bath*_ and fractional charge ω_*opt*_, as well as average energy Δ⟨*E*⟩_*opt*_ evaluated at both optimized temperature conditions (the Δ⟨*E*⟩ dependence with μ_*Bath*_ is reported in the Supporting Information). These profiles confirm that, for the bromine atom, the MSPP is valid within the μ_0_ ± 2 eV range. Beyond these values, however, the results from the MSPP diverge significantly from those predicted by the τ-CPEP. Of particular interest is the transferred charge, the τ-CPEP reveals a nearly perfect linear correlation between the transferred charge and the chemical potential of the reservoir within the – *I*_1_ > μ_*Bath*_ < – *A*_1_ range. On the other hand, the average energy behaves quadratically with μ_*Bath*_ under the same conditions. In contrast, the fractional charge and average energy computed using the MSPP approach are constrained within the thresholds of approximately −0.2 > ω_*Opt*_ < + 0.2 (blue profile, Figure 2c) and +2 > Δ*E*_*opt*_ < – 1 (blue profile, Figure 2d), respectively, within the same range of values of μ_*Bath*_.

After an exhaustive numerical analysis of over 10^4^ ficticious species with different combinations of the *I* and *A* values (see Figure S1 of Supporting Information), we found the linear fit in Figure 5c has the following exact expression,
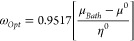
26

This remarkably straightforward equation perfectly encapsulates the charge transfer model based on the τ-CPEP framework presented here. What is particularly striking is that its form closely mirrors that derived from quadratic interpolation, but this time under the situation of a reservoir with no resistance to charge transfer (i.e., zero hardness). Even more intriguing is the intercept of this equation, which aligns with the μ_*Bath*_ = 0 condition, corresponding to the theoretical expression for charge transfer in an idealized sea of electrons as originally reported by Parr, von Szentpáli and Liu^15^—albeit scaled by a numerical factor, *i. e*., ω_max_ = −0.9517 μ^0^/η^0^. By substituting this result into the energy expression of eq 3, and working under the low temperature regime (consistent with the μ_*Bath*_ = 0 scenario), as shown in Figure 4, the following formula is obtained,
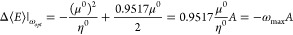
27

This result is particularly significant as it aligns with the widely accepted definition of the electrophilicity index. However, an additional term naturally emerges from the rigor of our theoretical development, which after simplification highlights the role of the electron affinity in the electrophilicity nature of the electronic systems. The computed electrophilicity index for the bromine atom, using eq 27, is −2.87 eV, a close value to the intercept estimator in Figure 5d (c = −2.86 eV). Introducing the ω_max_ definition in the density expression given in eq 5 and after a few manipulations, we obtain the definition for the local electrophilicity index,
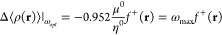
28which evidence the role of the Fukui function for the acceptation process in the local electrophilicity features of atoms and molecules and corresponds to the definition previously reported.^65,66^ Building on previous research suggesting that, for chemical reactivity purposes, the electron affinity should be constrained by a lower limit of zero,^67^ the electrophilicity in eq 27 reaches a maximum value of zero. This scenario corresponds to the case where the most favorable process involves the chemical species donating a full electron, necessitating that the coefficient in eq 26, as well as in eqs 27 and (28), be set to one. It is worth emphasizing that the precise value of this coefficient is inconsequential when comparing the reactivity features across a set of chemical species; likewise note that it is already nearly equal to one. Consequently, we propose that setting this parameter to one is not only more practical but also chemically consistent, without introducing any operational drawbacks.

The charge transfer process between an electronic species and its surroundings, as governed by the τ-CPEP, is qualitatively depicted in Figures S2a and S2b of the Supporting Information, which show the profiles of *S vs ω* and Δ⟨*E*⟩ *vs ω* for the lithium atom across several isotherms. Only the region of positive fractional charge is considered. Beginning at the point of maximum softness at *T* = *T*_*s*_Max_, the initial 0.12 units of charge are approximately transferred. As charge exchange progresses, heat is released, and according to the convexity analysis, roughly 0.16 units of charge can be transferred when the system reaches the *T* = *T*_C_υ__Max_ isotherm, with a subsequent loss of energy, approximately following a logarithmic decay. Beyond this point, further charge transfer causes a substantial temperature reduction, leading to a marked increase in softness until ω = 1/2 is transferred. Then, additional charge exchange accelerates the rate of variation and reduces softness. Thermal fluctuations mitigate this effect to some extent, but softness reaches its minimum value (near zero) when the next integer electron state is achieved, consistent with the MHP. Together, the MSPP, τ-CPEP and MHP statements offer a comprehensive and detailed framework for understanding the charge exchange behavior of an electronic species with its surroundings.

*Regioselectivity*. The theoretical findings presented here strongly suggest that heat exchange is the first step in chemical processes involving neutral (*N*-integer) species. Furthermore, eq 15 highlights that molecular regions where one Fukui function (local softness) significantly dominates over the other are primarily responsible for the changes in the density due to changes in the temperature. This indicates that regioselectivity originates during the heat exchange stage, preceding the actual charge exchange, since nucleophiles will show the largest changes in the density located in the region where *S*^–^(**r**) is substantially greater than *S*^+^(**r**), while electrophiles will show the largest changes in the density located in the region where *S*^+^(**r**) predominates.

Additionally, it is pertinent to consider the temperature dependent definition of the local softness descriptor,^50^
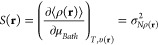
29which quantifies thermal fluctuations between the average electrons number and the average density. High values of local softness highlight regions where electron density would significantly deviate from equilibrium, facilitating density overlaps between reacting species when electrons are being transferred. Recently, it has been shown how local softness determines molecular polarizabilities using theoretic-informational arguments.^68^ The formula accounting for the representative three-state ensemble model can be found in the Supporting Information. Local softness decreases at low temperatures, suggesting that density overlap is more likely under high-temperature conditions. As discussed earlier, when a species acts as an electron donor, the dominant contribution to local softness comes from (**r**), while in electron-acceptor species, it is dictated by (**r**).

The theoretical findings presented here suggest that orbital overlap is promoted through the exchange of heat and electrons, with the zero-temperature definition of the Fukui function, or equivalently local softness, being the key factors in guiding regioselectivity throughout the whole heat and charge transfer processes.

It is important to note that the pioneering work of Yang and Parr^45^ that showed the relevance of the Fukui function to assess the importance of frontier orbital theory to describe regioselectivity, when this function is used in conjunction with the |Δμ| big is good principle is reinforced in the temperature dependent approach in two aspects. On one hand, the expression for the change in the electron density with respect to the temperature, eqs 12 and (15), underscores, through the Fukui function, the places that will suffer the largest changes at the onset of an interaction, before the charge transfer process occurs. On the other hand, the presence of the term given by eq 20 in the expression for the differential of the chemical potential, highlights that, indeed, through the change in the temperature one can also increase |Δμ|. This aspect is relevant given that it has been shown that this principle holds at temperatures beyond absolute zero.^37^

*Conclusions and perspectives.* The GE for electronic systems provides a rigorous theoretical framework for analyzing atomic and molecular responses to environmental perturbations. It builds upon the ensemble theorem developed by Perdew, Parr, Levy, and Balduz,^24^ extending it to finite temperature conditions. However, the theory indicates that at the temperatures at which reactions occur, fractional charge exchange only happens in discrete half-integer values, and the chemical potential equalization principle does not fully apply. These limitations are overcome in this work by introducing a vital concept: before charge transfer can happen, energy must be transferred as heat. This is not an alternative mechanism but rather the only viable one to drive the exchange of electronic charge between a neutral chemical species and its surroundings.

Our theoretical framework provides robust support for this new paradigm. Electrons undergo what can be described as a second-order phase transition, moving from an inert to a reactive state. Key indicators of this transition include: 1) the emergence of a maximum in the heat capacity, 2) large thermal fluctuations within the transition-phase zone, 3) the absorption of heat that decorrelates electrons, using up to 0.16 of the molecular *I*–*A* gap—this decorrelation energy may be viewed as an order parameter, and 4) some properties becoming independent of the microstates of the system.

Charge transfer occurs only after the heating stage and is accompanied by a general decrease in temperature as the system seeks the highest possible softness value that complies with the chemical potential equalization principle (τ-CPEP). Surprisingly, the resulting model of charge transfer simplifies to a linear dependence on the chemical potential of the reservoir. When extrapolated to μ_*Bath*_ = 0, this linear model provides solid theoretical support for both the electrophilicity index and local electrophilicity, with added correction terms that emphasize the significance of electron affinity and nucleophilic Fukui functions on these descriptors. In this framework, energy and charge flow together, with Fukui functions pinpointing the molecular sites most prone to this coupled exchange.

This work introduces a groundbreaking model of charge transfer that operates under temperature conditions far exceeding those typically considered in conventional chemistry. By exploring these unique electronic temperature values, this model opens up new pathways for a deeper understanding of chemical reactivity, addressing some unresolved yet crucial questions about the behavior of atoms and molecules. Key innovations and contributions of this model include:

1. Reactivity Descriptors at High Temperatures. The global, local and nonlocal temperature-dependent chemical reactivity descriptors have been so far computed under the zero-temperature limit approximation, disregarding the effects of absorbed heat. Evaluation of these thermal contributions could significantly improve the accuracy of chemical reactivity predictions and the understanding of chemical processes.
2. Refinement of the Electrophilicity Index. The Electrophilicity Index has been one of the most effective chemical reactivity descriptors within C-DFT. However, there has been extensive debate about the overemphasis on ionization potential in the current formula. By introducing a corrective term that naturally emerges from our theoretical framework, our revised electrophilicity index places greater emphasis on the role of electron affinity in electrophilicity. This refined formula promises to improve the accuracy of reactivity predictions.
3. Incorporating Excited States into Reactivity Calculations. Up to date, there is no general framework for quantifying the contributions of excited states to chemical reactivity, although advances for some circumstances have been recently reported.^69^ Given that the high temperatures required for charge transfer in our model may lead to considerable populations of higher-energy states, they would influence average properties and response functions.
4. Beyond Frontier Orbitals at Elevated Temperatures. In conventional one-particle states approaches, chemical reactivity is often attributed solely to frontier orbitals (HOMO and LUMO),^70,71^ while contributions from orbitals beyond this range are typically neglected. However, as temperature increases, both inner and outer orbitals relative to the HOMO and LUMO levels will become fractionally populated.^72^ These orbitals can meaningfully contribute to chemical reactivity.
5. Development of Local Temperature-Based Reactivity Models. Local temperature has been proposed as the scalar-field counterpart to global electronic temperature.^73,74^ It has been suggested that the local temperature concept carry valuable information about reactivity,^72,75−77^ and even of the reaction rate.^78^ By extending the Grand Ensemble (GE) model accounting local temperature as variable, it would be possible to develop a new space-dependent framework to study chemical reactivity at a higher level of resolution.
6. Time and Temperature-Dependent Reactivity Theory. The theoretical framework presented here provides a foundation for developing a time- and temperature-dependent approach to chemical reactivity. Although some advances in molecular responses experimenting electromagnetic perturbations^79−81^ or dynamical processes^82,83^ have been recently reported, by operating within the framework of nonequilibrium thermodynamics, this novel approach will enable the study of dynamic chemical processes, emphasizing photochemical reactions.

In conclusion, this work offers a groundbreaking description of charge transfer phenomena at the earliest stages of chemical interactions, utilizing the rigor of the fundamental framework of the GE of electronic systems. We hope that the theoretical and conceptual insights together with the numerical results presented here will inspire the broader chemistry community to explore and adopt the electronic-thermodynamic perspective on chemical reactivity, fostering a deeper understanding of these complex processes and their underlying mechanisms.

## Supporting Information Available

Working formulas for softness, heat capacity, average energy and local softness for an ensemble model constituted by the three fundamental states with *N*_0_, *N*_0_+1 and *N*_0_*-*1 electrons, plots of the *Slope* vs η and *Intercept* vs μ_e_/η profiles using values derived from the ω vs μ_*Bath*_ relation of a set of over 2.344 × 10^4^ artificial chemical species to corroborate the generalized linear behavior of the fractional charge ω with μ_*Bath*_ when the τ-CPEP operates, *S vs ω* and Δ⟨*E*⟩ *vs ω* profiles for the Lithium neutral atom, and a Python script to compute properties at transition phase point as well as imposing the MSPP and τ-CPEP principles (PDF)
