# Supplementary material for: Charge Transfer Is Promoted by Electronic Heat Exchange in Atoms and Molecules
Source: J Phys Chem Lett. 2025 Feb 24;16(9):2283–94. doi: 10.1021/acs.jpclett.4c03664 (PMC11891895; doi:10.1021/acs.jpclett.4c03664)
Supplement: Supplementary file 1 — jz4c03664_si_001.pdf [file jz4c03664_si_001.pdf]

Charge transfer is promoted by electronic heat exchange in atoms and molecules.

Marco Franco-Pérez*[1,2] and José L. Gázquez[2].

1 Universidad Nacional Autónoma de México, Cd. Universitaria, Facultad de Química, Ciudad de México 04510, México.
2 Universidad Autónoma Metropolitana-Iztapalapa, Departamento de Química, Av. San Rafael Atlixco 186, Ciudad de Mexico 09340, Mexico.

Corresponding author

Marco Franco-Pérez. Email: qimfranco@quimica.unam.mx

SUPPORTING INFORMATION

Form. Working formulas for S, C_{υ_r}, $\Delta\langle E\rangle$ and $S(\mathbf{r})$ as a function of the chemical potential of reservoir μ_{Bath} S2

Figure S1. a) Slope vs η and b) intercept vs μ_e/η profiles using values derived from the ω vs μ_{Bath} profiles of a set of over 2.344x10^4 artifitial chemical species: These species exhibit ionization potential values in the range of 3 to 18 eV and electron affinity values in the range of 0 to 5 eV S3

Figure S2. a) S *vs* ω and b) $\Delta\langle E\rangle$ *vs* ω profiles for the Lithium neutral atom $(I,\ A = 5.40,\ 0.62\ \text{eV})$, in the range $0 \le \omega \le 1$ S4

Routine. Python script to compute transition phase properties as well as the maximum softness path principle and the finite temperature chemical potential equalization principle S5

Form. Working formulas for S, C_{υ_r}, $\Delta\langle E\rangle$ and $S(\mathbf{r})$ as a function of the chemical potential of reservoir μ_{Bath}.

$$S=\beta\frac{e^{-\beta(I+\mu_{Bath})}+e^{\beta(A+\mu_{Bath})}+4e^{-\beta(I-A)}}{\left(1+e^{-\beta(I+\mu_{Bath})}+e^{\beta(A+\mu_{Bath})}\right)^{2}} \tag{ES1}$$

$$C_{\upsilon_r}=\beta\left(\frac{e^{-\beta(I+\mu_{Bath})}+e^{\beta(A+\mu_{Bath})}+4e^{-\beta(I-A)}}{T\left(1+e^{-\beta(I+\mu_{Bath})}+e^{\beta(A+\mu_{Bath})}\right)^{2}}\right)\left(\begin{array}{c}\dfrac{I^{2}\mathrm{e}^{-\beta(I+\mu_{Bath})}+A^{2}\mathrm{e}^{\beta(A+\mu_{Bath})}+4\left(\mu^{0}\right)^{2}\mathrm{e}^{-\beta(I-A)}}{e^{-\beta(I+\mu_{Bath})}+e^{\beta(A+\mu_{Bath})}+4e^{-\beta(I-A)}}\\ -\left(\dfrac{-I\mathrm{e}^{-\beta(I+\mu_{Bath})}+A\mathrm{e}^{\beta(A+\mu_{Bath})}+4\mu^{0}\mathrm{e}^{-\beta(I-A)}}{e^{-\beta(I+\mu_{Bath})}+e^{\beta(A+\mu_{Bath})}+4e^{-\beta(I-A)}}\right)^{2}\end{array}\right)$$

(ES2)

$$\Delta\langle E\rangle=\frac{Ie^{-2\beta(\mu_{Bath}-\mu_0)}-A}{1+e^{-2\beta(\mu_{Bath}-\mu_0)}+e^{-\beta(A+\mu_{Bath})}} \tag{ES3}$$

$$S(\mathbf{r})=\beta\frac{f^{-}(\mathbf{r})e^{-\beta(I+\mu_{Bath})}+f^{+}e^{\beta(A+\mu_{Bath})}+4f^{0}e^{-\beta(I-A)}}{\left(1+e^{-\beta(I+\mu_{Bath})}+e^{\beta(A+\mu_{Bath})}\right)^{2}} \tag{ES4}$$

Figure S1. a) Slope vs η and b) intercept vs μ_e/η profiles using values derived from the ω vs μ_{Bath} profiles of a set of over 2.344x10^4 artifitial chemical species: These species exhibit ionization potential values in the range of 3 to 18 eV and electron affinity values in the range of 0 to 5 eV.

a)

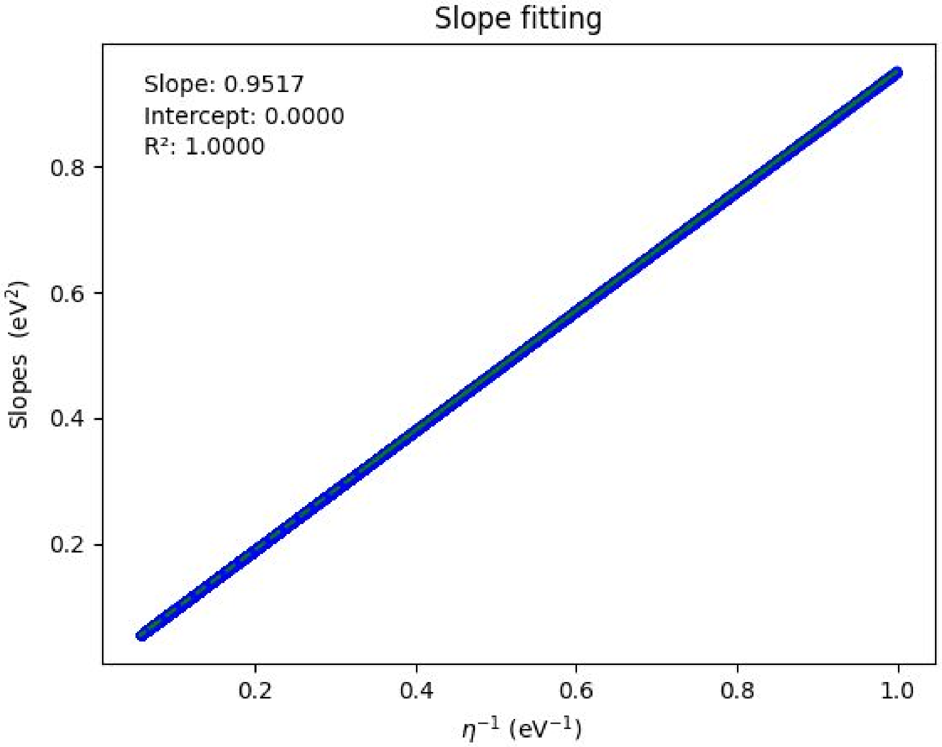

b)

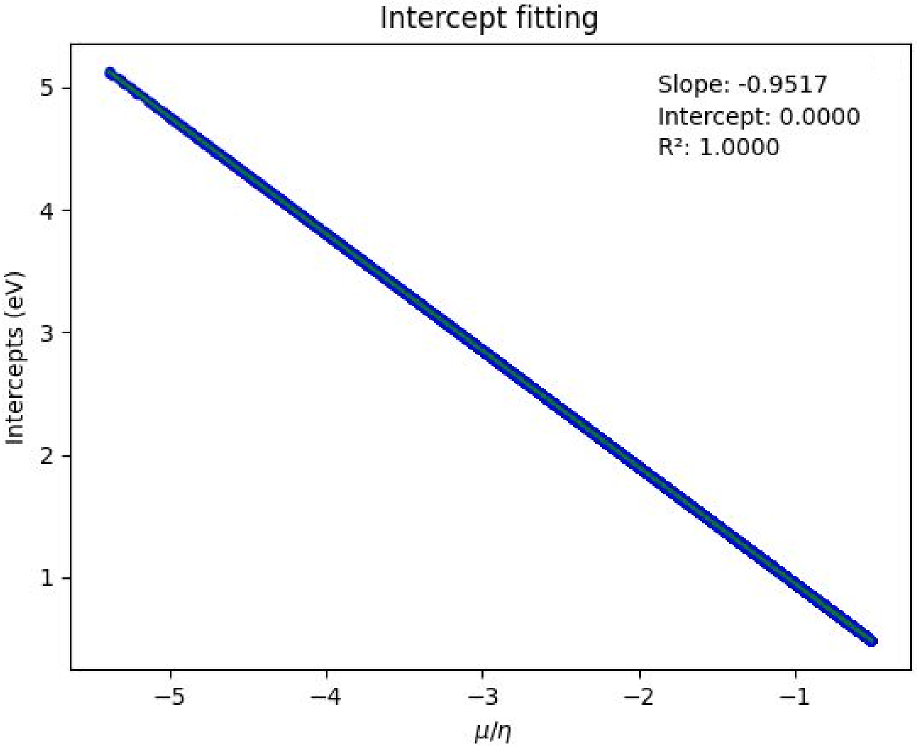

Figure S2. a) S *vs* ω and b) $\Delta\langle E\rangle$ *vs* ω profiles for the Lithium neutral atom $(I,\ A = 5.40,\ 0.62\text{ eV})$, in the range $0 \le \omega \le 1$. Energy units in eV. The same set of temperatures and code of colors were used to build both profiles. Discontinuous black lines are used to qualitatively represents the softness-temperature and energy-temperature evolutions during charge transfer, respectively. Dark and red dots are used to indicate the $\omega = 0.12$ and $\omega = 0.16$ values expected to be observed at the $T = T_{S\_\mathrm{Max}}$ and $T = T_{\mathrm{C}_\upsilon\_\mathrm{Max}}$ isotherms.

a)

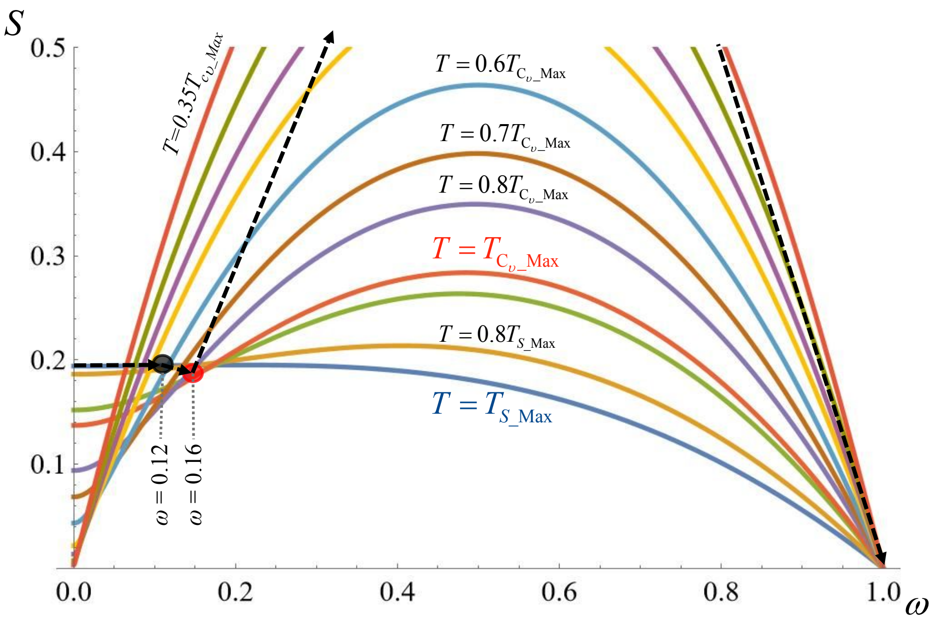

b)

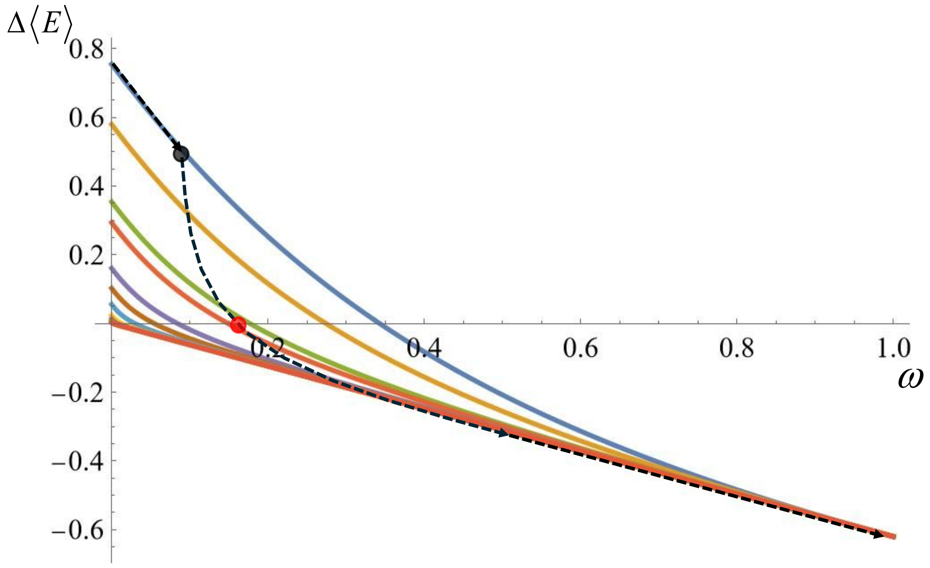

Routine

Python script to compute transition phase, maximum softness path principle and the finite temperature chemical potential equalization principle related properties.

```
import numpy as np
from scipy.optimize import minimize_scalar

class ChargeTransferModel:
    """
    A model to study charge transfer processes between atoms/molecules using
the principles of
    soft maximization based on chemical potential, ionization energy, and
electron affinity.
    """

    k_B = 0.000086173324  # Boltzmann constant in eV/K

    def __init__(self, mu_reservoir, ionization_energy, electron_affinity,
verbose=False):
        """
        Initialize the ChargeTransferModel class with required parameters.

        Parameters:
        ----------
        mu_reservoir : float
            Chemical potential of the reservoir (in eV).
        ionization_energy : float
            Ionization energy of the species (in eV).
        electron_affinity : float
            Electron affinity of the species (in eV).
        verbose : bool, optional
            If True, outputs detailed information during optimization (default
is False).
        """
        self.mu_reservoir = mu_reservoir
        self.ionization_energy = ionization_energy
        self.electron_affinity = electron_affinity
        self.mu_0 = -(self.ionization_energy + self.electron_affinity) / 2
        self.delta_mu = self.mu_reservoir - self.mu_0
        self.hardness = self.ionization_energy - self.electron_affinity
        self.verbose = verbose

    def compute_critical_properties(self):
        """Calculate critical properties in the transition zone."""
        eta_0 = self.hardness
        t_cv_max = (0.18834 / self.k_B) * eta_0  # Maximum temperature for Cv
        t_s_max = (0.34175 / self.k_B) * eta_0  # Maximum temperature for S
        decorrelation_energy = 0.158 * eta_0  # Decorrelation energy
        return t_cv_max, t_s_max, decorrelation_energy

    def thermal_beta(self, temperature):
        """Calculate the inverse thermal energy (1/kT)."""
        return 1 / (self.k_B * temperature)

    def softness_function(self, temperature):
        """
        Define the softness function that will be optimized.

        Parameters:
        ----------
        temperature : float
            Temperature value for which the softness is calculated.

        Returns:
        -------
```

```
        float
            Negative of softness for minimization purposes.
        """
        beta = self.thermal_beta(temperature)

        exp_ionization = np.exp(-beta * (self.ionization_energy +
self.mu_reservoir))
        exp_affinity = np.exp(beta * (self.electron_affinity +
self.mu_reservoir))
        exp_difference = np.exp(-beta * (self.ionization_energy -
self.electron_affinity))

        term1 = exp_ionization + exp_affinity + 4 * exp_difference
        term2 = (1 + exp_ionization + exp_affinity) ** -2

        return -beta * term1 * term2  # Negated for maximization

    def optimize_softness(self):
        """Optimize the softness function to find the optimal temperature."""
        result = minimize_scalar(self.softness_function, method='bounded',
bounds=(1, 1e5))
        return result.x, -result.fun  # Return optimal temperature and the
maximized softness

    def equalize_chemical_potential(self):
        """
        Minimize the difference between the electronic chemical potential and
the reservoir's chemical potential.

        Returns:
        -------
        float
            Optimal temperature at which chemical potential equalization
occurs.
        float
            Minimum value of the chemical potential difference.
        """

        def target_function(temperature):
            return abs(self.compute_electronic_potential(temperature) -
self.mu_reservoir)

        result = minimize_scalar(target_function, method='bounded', bounds=(1,
1e5))
        return result.x, result.fun

    def calculate_charge(self, temperature):
        """
        Calculate the fractional charge transferred between the species and
the reservoir.

        Parameters:
        ----------
        temperature : float
            Temperature value at which charge is calculated.

        Returns:
        -------
        float
            Fractional charge transferred.
        """
        beta = self.thermal_beta(temperature)
        numerator = (1 - np.exp(-2 * beta * self.delta_mu))
        denominator = (1 + np.exp(-2 * beta * self.delta_mu) + np.exp(
            -beta * (self.electron_affinity + self.mu_reservoir)))
        return numerator / denominator

    def compute_electronic_potential(self, temperature):
```

```
        """
        Calculate the electronic chemical potential based on temperature.

        Parameters:
        ----------
        temperature : float
            Temperature value at which the potential is calculated.

        Returns:
        -------
        float
            Electronic chemical potential at the given temperature.
        """
        charge = self.calculate_charge(temperature)
        beta = self.thermal_beta(temperature)
        alpha = np.sqrt(charge ** 2 + 4 * (1 - charge ** 2) * np.exp(-beta *
self.hardness))
        return self.mu_0 + 0.5 * self.hardness * charge / alpha

    def run_optimization(self):
        """
        Run the optimization process for maximizing softness and equalizing
chemical potential.

        Returns:
        -------
        dict
            A dictionary containing critical properties, optimization results,
and equalization results.
        """
        # Calculate critical properties
        t_cv_max, t_s_max, decorrelation_energy =
self.compute_critical_properties()

        # Maximize softness
        optimal_temperature, max_softness = self.optimize_softness()
        transferred_charge_softness =
self.calculate_charge(optimal_temperature)
        electronic_potential =
self.compute_electronic_potential(optimal_temperature)

        # Equalize chemical potential
        equalization_temperature, _ = self.equalize_chemical_potential()
        transferred_charge_equalization =
self.calculate_charge(equalization_temperature)
        equalized_potential =
self.compute_electronic_potential(equalization_temperature)
        softness_value = self.softness_function(equalization_temperature)

        # Package results
        results = {
            'critical_properties': {
                'T_cv_max': round(t_cv_max, 2),
                'T_s_max': round(t_s_max, 2),
                'Decorrelation_energy': round(decorrelation_energy, 3),
            },
            'optimization_results': {
                'optimal_temperature': round(optimal_temperature, 2),
                'transferred_charge': round(transferred_charge_softness, 3),
                'electronic_softness': round(max_softness, 4),
                'electronic_potential': round(electronic_potential, 3),
            },
            'equalization_results': {
                'equalization_temperature': round(equalization_temperature,
2),
                'transferred_charge': round(transferred_charge_equalization,
3),
                'equalized_potential': round(equalized_potential, 3),
```

```
                'softness_value': round(softness_value, 3),
            }
        }

        if self.verbose:
            print(
                f"***All results in eV, K***\n\n"
                f"Critical Properties:\n "
                f"T_cv_max = {round(t_cv_max, 2)},\n "
                f"T_s_max = {round(t_s_max, 3)},\n "
                f"Decorrelation_energy = {round(decorrelation_energy, 3)}\n")
            print(
                f"Softness Maximization Results:\n "
                f"Optimal Temperature = {round(optimal_temperature, 2)},\n "
                f"Transferred Charge = {round(transferred_charge_softness,
3)},\n "
                f"Electronic Chemical Potential = {round(electronic_potential,
2)},\n "
                f"Electronic Softness = {round(max_softness, 4)}\n")
            print(
                f"CP Equalization Results:\n "
                f"Equalization Temperature = {round(equalization_temperature,
2)},\n "
                f"Transferred Charge = {round(transferred_charge_equalization,
3)},\n "
                f"Equalized Chemical Potential = {round(equalized_potential,
2)},\n "
                f"Electronic_softness: {round(-softness_value, 4)} eV")

        return results


# Example usage for charge transfer between a carbon atom immersed in bromine
atoms
if __name__ == "__main__":
    ionization_energy = 11.26  # eV for Carbon
    electron_affinity = 1.28  # eV for Carbon
    mu_reservoir = -7.59  # eV for Bromine

    # Instantiate the ChargeTransferModel class
    optimizer = ChargeTransferModel(mu_reservoir, ionization_energy,
electron_affinity, verbose=True)

    # Run the optimization
    results = optimizer.run_optimization()
```
